# FPGA-Based Implementation and Synchronization Design of a New Five-Dimensional Hyperchaotic System

**DOI:** 10.3390/e24091179

**Published:** 2022-08-24

**Authors:** Ya Wang, Xinyu Li, Xiaodong Li, Yerui Guang, Yanan Wu, Qun Ding

**Affiliations:** 1 Electronic Engineering College, Heilongjiang University, Harbin 150080, China; 2 Beijing Aerospace Institute of Automatic Control, Beijing 100854, China

**Keywords:** complex chaotic system, nonlinear feedback control, chaotic synchronization, FPGA, secure communication

## Abstract

Considering the security of a communication system, designing a high-dimensional complex chaotic system suitable for chaotic synchronization has become a key problem in chaotic secure communication. In this paper, a new 5-D hyperchaotic system with high order nonlinear terms was constructed and proved to be hyperchaotic by dynamical characterization characteristics, the maximum Lyapunov exponent was close to 2, and there was a better permutation entropy index, while a valid chaotic sequence could be generated in three cycles in the FPGA (Field Programmable Gate Array)-based implementation. A multivariable nonlinear feedback synchronous controller based on FPGA was proposed to design and implement synchronization of high order complex hyperchaotic systems. The results show that the error signal converged to 0 rapidly under the effect of the nonlinear feedback synchronous controller. This lays the foundation for the synchronization of high order complex chaotic systems.

## 1. Introduction

With the increasing demand for information security, secure communication technology research has become particularly important, and the application of chaos theory in secure communication has attracted increasing attention. Chaotic synchronization is a significant part of chaotic secure communication research. The transceiver system for chaotic secure communication has been extended from a low-dimensional chaotic system to a high-dimensional chaotic system. Creating high-dimensional complex chaotic systems and achieving chaotic synchronization have become the key in the research of chaotic secure communication.

In 1963, the American meteorologist E. N. Lorenz observed chaos in his atmospheric studies, opening the way for future exploration of chaos [1]. Chaos has favorable randomness and it is widely used in various fields such as secure communication [2], medical image processing [3,4,5], biomedical science [6,7], and finance [8,9,10]. In 1979, Otto Rössler proposed the first hyperchaotic system with two or more attractors and positive Lyapunov exponents, its phase orbitals can be separated in multiple directions, and the algebraic structure and dynamical behavior are more complex, confidential, and impenetrable than ordinary low-dimensional chaotic systems, with greater potential for research and development.

In [11], Chengqun zhou et al. constructed a four-dimensional hyperchaotic system based on the Lorenz system with the properties of stability, periodicity, multiple coexisting attractors, multiplicative period, and Hopf bifurcation, the maximum Lyapunov exponent being 0.4934. Li et al. proposed a new four-dimensional hyperchaotic system with exponential terms, the basic dynamical properties and chaotic behavior of the new attractor were analyzed. The results showed that the new hyperchaotic system has an equilibrium point or a single equilibrium point. The maximum Lyapunov exponent of the hyper-chaotic system is 0.1782 [12]. Sundarapandian et al. proposed a new multi-stable four-dimensional hyperchaotic system and confirmed the special properties of the new system such as multi-stability of coexisting attractors by bifurcation diagram, phase diagram, and dynamics analysis [13]. Bouteghrine et al. proposed a new multidimensional chaotic system with multiple parameters and nonlinear terms, and a two-phase algorithm was proposed to study the chaotic behavior using bifurcation and Lyapunov exponential theory [14].

Currently, there is little research on high-dimensional chaotic systems with high order terms. Meanwhile, the maximum Lyapunov exponent of most hyperchaotic systems does not exceed 1. For hyperchaotic systems, the larger the Lyapunov exponent, the better the performance of the system. Thus, it is necessary to investigate high-dimensional, high order hyperchaotic systems with larger Lyapunov exponents.

Compared with traditional programmable devices, FPGA (Field Programmable Gate Array) is of great interest to engineers because of the excellent properties such as design flexibility, high integration, and high-speed parallel processing, and is gradually occupying an increasing share in today’s digital information market. Yuan et al. implemented a chaotic circuit for a new Chen-like system by introducing the product and square terms of variable coefficients and using 32-bit fixed-point operations with the help of the Quartus II 13.0 platform from ALTERA [15]. Sun et al. similarly used the Lorenz system as the object and completed the chaotic system with a modular design approach in the form of data processing by floating-point operations through a hardware description language [16]. Xue et al. completed a synchronization and corresponding confidential video communication system based on the hyperchaotic system using FPGA [17]. Liu designed and implemented the computing architecture of the Qi hyperchaotic system based on FPGA technology, which required 27 clock cycles to generate a new valid chaotic iteration value [18]. Tang et al. designed and implemented the computing architecture of the Chen hyperchaotic system based on FPGA technology, which required five clock cycles to generate a new valid chaotic iteration value [19]. In the investigation of FPGA-based implementation of chaotic systems, numerous clock cycles to generate a valid chaotic sequence, in the application to the field of secure communication, reduce the efficiency of the operation of encryption and decryption of plaintext data, thus reducing the overall efficiency.

Chaotic systems are widely used in the field of secure communication because of their high initial value sensitivity and unpredictability, but they have also been considered unsynchronizable for a long time. It was not until 1990 that T.L. Pecora and L.M. Carroll first proposed drive-response synchronization and observed synchronization in circuits, opening the door for the study of chaotic synchronization methods and their applications [20].

Several chaotic synchronization methods have been proposed, including adaptive synchronization control [21], backstepping control [22], nonlinear feedback control [23], and drive-response synchronization control [24]. Moon Sungju et al. investigated the self-synchronization problem of high-dimensional Lorenz systems and showed that satisfactory synchronization results could not be obtained when the dimensions of the drive and response systems were different [25]. In [26], Dan Li et al. used a drive-response synchronization control method to achieve two 6-vortex chaotic attractor synchronizations based on the recent multi-vortex chaotic attractor synchronization theory. Liu Yangzheng et al. constructed a new four-dimensional hyperchaotic Liu system based on the three-dimensional Liu system. The chaotic synchronization of this hyperchaotic system was achieved by using a nonlinear feedback control method. Based on the stability theory of the system, the structure of the nonlinear feedback controller and the range of values of the feedback control gain when the system reaches chaotic synchronization were obtained [27]. Compared with other synchronization methods, the nonlinear feedback synchronization control does not need to decompose the system and is more suitable for complex high-dimensional chaotic systems.

In this paper, a five-dimensional chaotic system with cubic nonlinear terms is proposed and implemented by FPGA. The Lyapunov exponential spectrum, bifurcation diagram, permutation entropy, and heterogeneity of the new chaotic system are analyzed and the resulting sequences are tested with the NIST SP800-2 standard. A new five-dimensional hyperchaotic system based on FPGA is designed and implemented. In conjunction with the Lyapunov stability theory, a multivariate non-linear feedback synchronous controller is designed and analyzed. Finally, the FPGA hardware design is completed and validated at the board level using the Vivado development platform and the ARTIX-7 development board.

The subsequent sections of this paper are organized as follows: Section 2 describes the new 5-D hyperchaotic system used in this paper. The proposed non-linear feedback synchronous control by FPGA is described in Section 3. Section 4 presents the simulation results. Conclusions are drawn in Section 5.

## 2. New 5-Dimensional Hyperchaotic System and Its Dynamical Properties

### 2.1. Theory of Hyperchaotic System

In 1963, in his work on the effects of atmospheric convection on climate, the American meteorologist Lorenz used Newtonian mechanics to establish a set of nonlinear differential equations, which can be expressed as follows:

(1) $$\begin{array}{l} \dot{x}=\alpha (y-x)\\ \dot{y}=\gamma x-y-\mathrm{xz}\\ \dot{z}=\mathrm{xy}-\beta z \end{array}$$

In 2005, Qi et al. proposed the Qi hyperchaotic system, which contains three subsystems, Lorenz hyperchaotic, Chen hyperchaotic and Lü hyperchaotic, which can produce more complex dynamical properties with attractors showing biplanarity and a larger trajectory traversal range in phase space [24]. Qi hyperchaotic can be expressed as:

(2) $$\begin{array}{l} \dot{x}=\alpha (y-x)+\mathrm{yzw}\\ \dot{y}=\beta (x+y)-\mathrm{xzw}\\ \dot{z}=-\gamma z+\eta \mathrm{xyw}\\ \dot{w}=-\sigma w+\mathrm{xyz} \end{array}$$

where x, y, z, w are state vectors. When α=50, β=4, γ=13, σ=20, η=4, there exists two positive Lyapunov exponents and the system exhibits a hyperchaotic state.

By simultaneously adding dimensionality and nonlinear terms to this system, this paper proposes a new 5-D chaotic system with cubic nonlinear terms, which can be defined as:

(3) $$\begin{array}{l} \dot{x}=\mathrm{ay}-\mathrm{bx}+\mathrm{cyz}+\mathrm{yzw}\\ \dot{y}=\mathrm{dx}+\mathrm{ly}-\mathrm{xz}-\mathrm{fv}-\mathrm{xzw}\\ \dot{z}=-{\mathrm{hz}+y}^{2}+\mathrm{xyw}\\ \dot{w}=-\mathrm{jyz}-\mathrm{pw}+\mathrm{xyz}\\ \dot{v}=k(x+v) \end{array}$$

where x, y, z, w, v represent state vectors and a, b, c, d, l, f, h, j, p, k represent constant parameters.

### 2.2. Dynamical Properties Analysis

#### 2.2.1. Dissipativity

In relation to system (3) it is known that:

(4) $$\nabla V=\;\frac{\partial \dot{x}}{\partial x}\;+\;\frac{\partial \dot{y}}{\partial y}\;+\;\frac{\partial \dot{z}}{\partial z}\;+\;\frac{\partial \dot{w}}{\partial w}\;+\;\frac{\partial \dot{v}}{\partial v}\;=\;-b+l-h-p+k$$

When ∇V=−b+l−h−p+k < 0, the chaotic system is dissipative and converges exponentially, the rate of phase space convergence can be calculated to be:

(5) $$\begin{array}{l} \frac{\mathrm{dv}}{\mathrm{dt}}=-(b-l+h+p-k)V\\ {V=e}^{-(b-l+h+p-k)}\frac{\mathrm{dv}}{\mathrm{dt}} \end{array}$$

Namely, this chaotic system converges to a volume $V_{0}$ at moment t to $e^{-(b-l+h+p-k)}$. When time t tends to infinity, all the orbits of the system converge to a subset of zero measure, i.e., generating chaotic attractors.

#### 2.2.2. Lyapunov Exponents and Bifurcation Diagram

**Figure 1:**
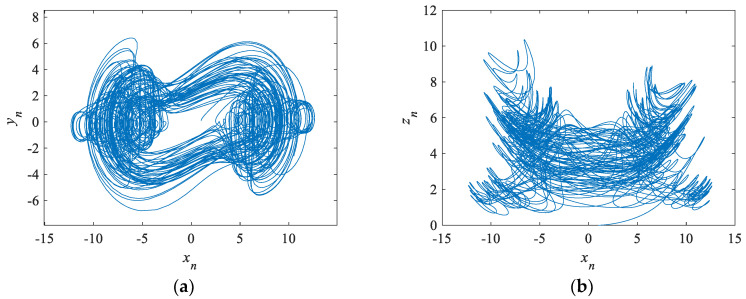

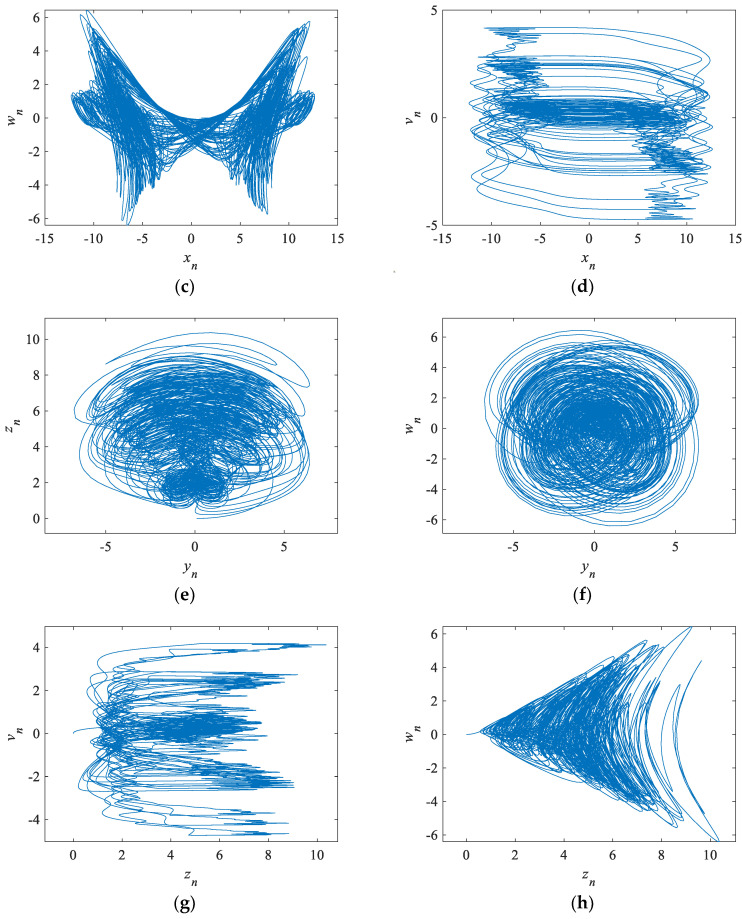
Phase diagrams of a hyperchaotic system. (**a**) Phase diagram of the x-y plane. (**b**) Phase diagram of the x-z plane. (**c**) Phase diagram of the x-w plane. (**d**) Phase diagram of the x-v plane. (**e**) Phase diagram of the y-z plane. (**f**) Phase diagram of the y-w plane. (**g**) Phase diagram of the z-v plane. (**h**) Phase diagram of the z−w plane.

Taking the parameters as a = 14, b = 0.5, c = 2, d = 2, l = 6, f = 4.5, h = 3, j = 0.5, p = 15, k = 0.423, for the initial value of the chaotic system ${(x}_{0}{,y}_{0}{,z}_{0}{,w}_{0}{,v}_{0})=(1,0.25,2,-1,1.5)$, the Lyapunov exponents of the system can be obtained as ${\mathrm{LE}}_{1}=1.9380$, ${\mathrm{LE}}_{2}=0.1391$, ${\mathrm{LE}}_{3}=-0.0084$, ${\mathrm{LE}}_{4}=-2.3637$, ${\mathrm{LE}}_{5}=-11.1328$, indicating the chaotic system is a hyperchaotic system. The partial phase diagrams of this hyperchaotic system are shown in Figure 1. 

**Figure 2:**
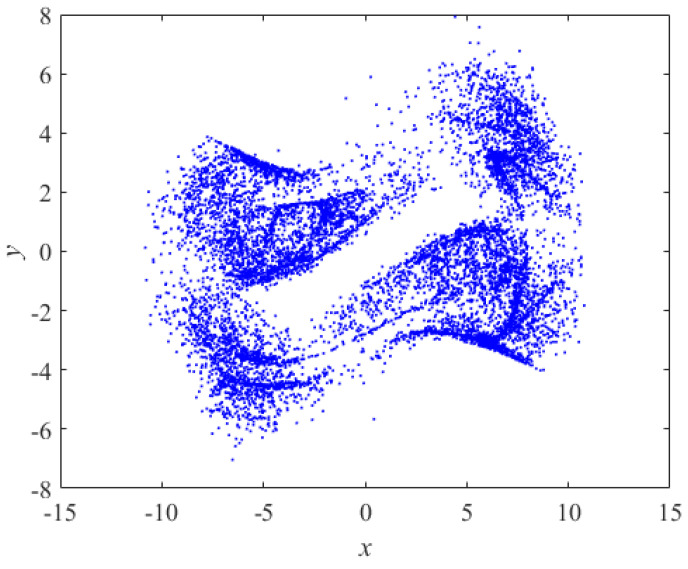
Cross-sectional view of Poincaré.

The Poincaré cross section converts the trajectory of a dynamical system to its intersection with the cross section to be studied. The continuous trajectory of the phase space is presented as some discrete points in the cross-section, the changes of the system morphology can be judged by these discrete points: the quasi-periodic motion is presented as a closed curve; the periodic motion is presented as a small number of discrete points; the chaotic motion is presented as aggregated and dense points. Figure 2 shows the x-y cross section selection at z = 5. It can be observed that the points are clustered and dense, indicating that the system is in a hyperchaotic state.

**Figure 3:**
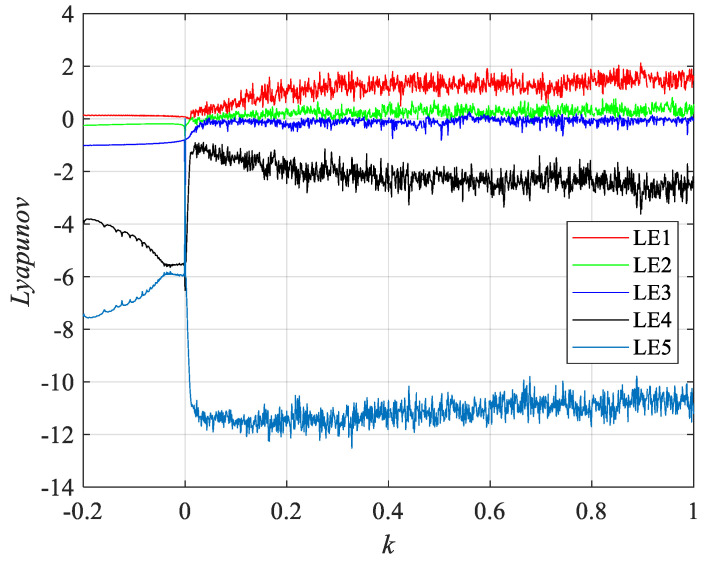
Spectrum of Lyapunov exponents.

**Figure 4:**
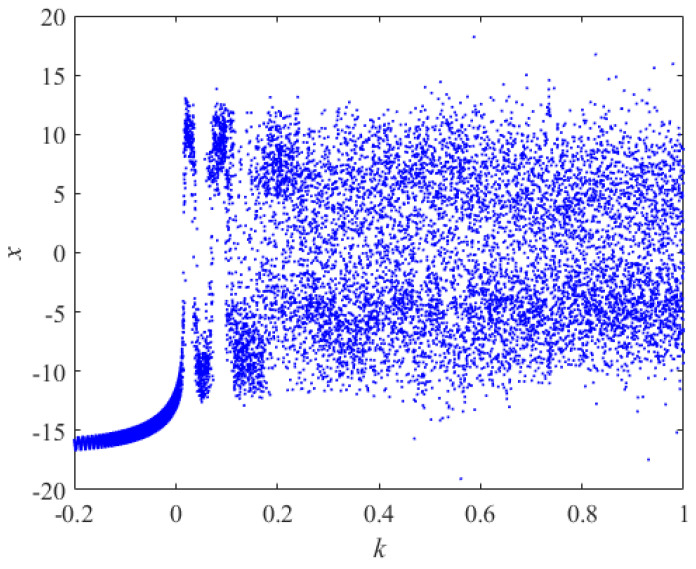
Bifurcation diagram of x with k.

**Figure 5:**
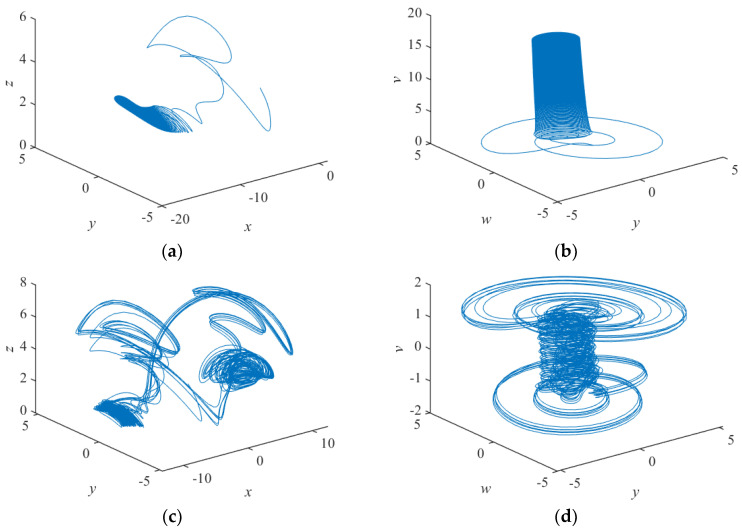

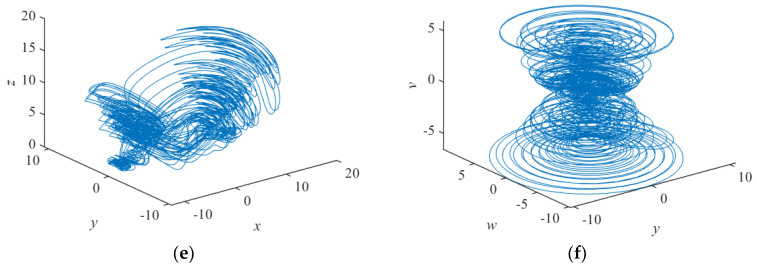
Phase diagrams of various attractor morphologies (**a**) x-y-z plane phase diagram of periodic attractor, (**b**) y-v-w plane phase diagram of periodic attractor, (**c**) x-y-z plane phase diagram of chaotic attractor, (**d**) y-v-w plane phase diagram of chaotic attractor, (**e**) x-y-z plane phase diagram of hyperchaotic attractor, (**f**) y-v-w plane phase diagram of hyperchaotic attractor.

Such that a = 14, b = 0.5, c = 2, d = 2, l = 6, f = 4.5, h = 3, j = 0.5, p = 15, 0.2≤k≤1, for the initial value ${(x}_{0}{,y}_{0}{,z}_{0}{,w}_{0}{,v}_{0})=(1,0.25,2,-1,1.5)$, Figure 3 and Figure 4 depict the variation of the Lyapunov exponent with the parameter k and the corresponding bifurcation diagram respectively. With the increase of k, the attractor exhibits a different form. For k = −0.095, one Lyapunov exponent is equal to zero, and the rest of the Lyapunov exponent is negative, representing that the attractor is in the form of a periodic attractor. For k = 0.055, there is a positive Lyapunov exponent and there exists a Lyapunov exponent equal to zero, indicating that the system is chaotic. For k = 0.423, there are two positive Lyapunov exponents at this point, suggesting that the system is hyperchaotic. The bifurcation diagram also shows the system switching between the periodic, chaotic, and hyperchaotic state with the change of k. The specific values are shown in Table 1. The corresponding three-dimensional phase diagrams of the system are shown in Figure 5.

Table 2 shows the comparison of the maximum Lyapunov exponent of the hyperchaotic system proposed in this paper with several hyperchaotic systems in the literature. As can be seen from Table 2, the maximum Lyapunov exponent of the five-dimensional hyperchaotic system proposed in this paper is 1.9380, which is much higher than the maximum Lyapunov exponent of other hyperchaotic systems, indicating that the system proposed in this paper has a superior performance compared with other systems.

#### 2.2.3. Randomness and Initial Value Sensitivity

The NIST system test standard was proposed by the National Institute of Standardized Technology (NIST) and is widely used in sequential randomized testing. The output sequence is tested with the NIST-SP800-2 test suite and the data length is 1 × 10^6^ bits. The significance level was determined to be 0.01. When *p*-value > 0.01, the test is qualified, and the results are shown in Table 3. It could be found that the output sequence of the new chaotic system passed the 16 NIST tests, and the results showed that the obtained sequence had good randomness.

Initial value sensitivity is one of the characteristics of a non-linear dynamical system and a criterion for measuring the stochasticity of the system. According to the established dynamical equations, changing the initial value of the system will change the dynamical behavior dramatically. When the difference between the two initial conditions is small, the dynamic behavior will initially remain the same or behave similarly, and with the increase of time, the dynamic behavior will be significantly different.

**Figure 6:**
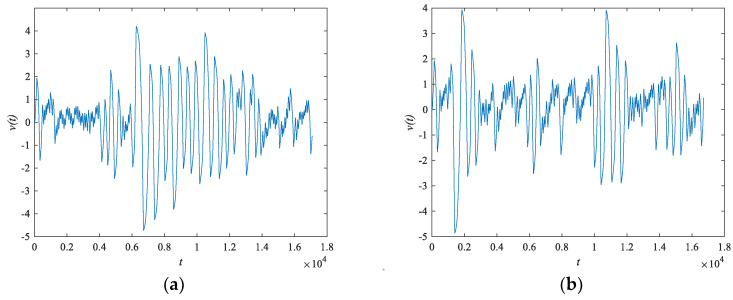
Time-series diagram comparison of v(t) (**a**) ${(x}_{0}{,y}_{0}{,z}_{0}{,w}_{0}{,v}_{0})=(1,0.1,0,0,0)$ sequence; (**b**) ${(x}_{0}{,y}_{0}{,z}_{0}{,w}_{0}{,v}_{0})=(1,0.1,0,0,0.0001)$ sequence.

V(t) for example, the output sequence, is shown in Figure 6a for the initial value ${(x}_{0}{,y}_{0}{,z}_{0}{,w}_{0}{,v}_{0})\;=\;(1,0.1,0,0,0)$ and in Figure 6b for ${(x}_{0}{,y}_{0}{,z}_{0}{,w}_{0}{,v}_{0})\;=\;(1,0.1,0,0,0.0001)$. Observing the two figures, it is clear that even if the initial value is only modified by 0.0001, the output trajectory remains similar only at the beginning, and the trajectory is completely distinct as the time and number of iterations increase. This indicates that the system has favorable initial value sensitivity.

#### 2.2.4. Permutation Entropy

The complexity of a chaotic system refers to the degree to which a chaotic sequence is close to a pseudo-random sequence using a correlation algorithm. The larger the complexity value, the closer the sequence is to a random sequence, and the higher the corresponding security. The permutation entropy algorithm belongs to one of the algorithms for calculating the complexity of a chaotic system, and the permutation entropy algorithm can be expressed as follows:

(a) There exists a time series of length N x(1),x(2),x(3),…,x(N), embedding dimension m with time delay t for phase space reconstruction.
(b) The reconstructed subsequence can be expressed as X(i), where X(i)=x(i),x(i+t),…,x(i+(m−1)t), and the reconstructed matrix Y is obtained, which can be expressed as:
  (6) $$Y=\left[\begin{array}{cccc} x(1) & x(1+t) & L & x(1+(m-1)t)\\ x(2) & x(2+t) & L & x(2+(m-1)t)\\ x(j) & x(j+t) & L & x(j+(m-1)t)\\ \begin{array}{c} M\\ x(i) \end{array} & \begin{array}{c} M\\ x(i+t) \end{array} & \begin{array}{l} L\\ L \end{array} & \begin{array}{c} M\\ x(i+(m-1)t) \end{array} \end{array}\right]$$
  where i=N−(m−1)t, each row of the matrix Y is a reconstructed component, and there are i reconstructed components. By reordering each reconstructed component X(i) in ascending order, the column indices of the positions of the elements in the vector are obtained to form a set of symbolic sequences $S(l)=\left\{j_{1}{,j}_{2},\ldots {,j}_{m}\right\},l=1,2,\ldots ,i$, and i≤m!, thus are mapped to $\left\{j_{1}{,j}_{2},\ldots {,j}_{m}\right\}$.
(c) Calculating the number of occurrences of each symbol sequence divided by the total number of occurrences of m! different symbol sequences as the probability of the occurrence of that symbol sequence, it can be expressed as $\left\{P_{1}{,P}_{2},\ldots {,P}_{i}\right\}$.
(d) The entropy of the permutation of the time series can be expressed as:
  (7) $$H(m)=-\sum_{j=1}^{i}P_{j}\ln(P_{j})$$
(e) where $P_{i}=1/m!$, that is, each symbol has an equal probability, at this point the complexity of the time series is the highest, the permutation entropy is the largest, the permutation entropy is ln(m!), and for the convenience of representation, H(m) is normalized and expressed as follows:
  (8) $$0\leq \frac{H(m)}{\ln(m!)}\leq 1$$

Taking the x(t) series as an example, the proposed system time series in this paper was compared with other literature, and the results are shown in Table 4.

As can be seen from Table 4, it can be concluded that the chaotic system proposed in this paper produces a higher entropy of sequence permutation, i.e., a higher complexity of the sequence, which can be effectively applied to improve security performance in areas such as secure communication.

In summary, a five-dimensional hyperchaotic system containing three nonlinear terms is proposed. The analysis of dynamics characteristics shows that the maximum Lyapunov exponent of the system can reach 1.9380, and from the analysis of permutation entropy it can be concluded that the new hyperchaotic system has a better permutation entropy exponent of 0.7042, which indicates that its complexity is higher and the corresponding security is higher; The proposed hyperchaotic system contains five signal variables and ten system parameters, multiple signal variables and system parameters make the key space larger, and its application to the field of confidential communication and image encryption can greatly enhance the security of the system and improve the confidentiality of communication.

## 3. FPGA-Based Hyperchaotic Synchronization Design

### 3.1. Hyperhaotic Synchronization Algorithm Design

Since FPGA can only process discrete digital signals, 5-D hyperchaotic systems need to discretize. Currently, the main discretization methods comprise the Runge–Kutta and Euler discretization. The Runge–Kutta method offers higher accuracy, while the hardware implementation is more difficult and consumes more hardware resources. In contrast, the Euler discretization method provides a relatively well-balanced compromise between hardware resource consumption and accuracy. Considering the large hardware overheads associated with longer data formats, this paper adopts a 24-bit fixed-point format, of which the higher 6 bits are the integer parts, where the highest bit is the sign bit and the lower 18 bits are the fractional parts.

The Euler discrete master system can be formulated as:

(9) $$\begin{array}{l} x(n+1)=[\mathrm{ay}(n)-\mathrm{bx}(n)+\mathrm{cy}(n)z(n)+y(n)z(n)w(n)]\times T+x(n)\\ y(n+1)=[\mathrm{dx}(n)+\mathrm{ly}(n)-x(n)z(n)-\mathrm{fv}(n)-x(n)z(n)w(n)]\times T+y(n)\\ z(n+1)=[-{\mathrm{hz}(n)+y(n)}^{2}+x(n)y(n)w(n)]\times T+z(n)\\ w(n+1)=[-\mathrm{jy}(n)z(n)-\mathrm{pw}(n)+x(n)y(n)z(n)]\times T+w(n)\\ v(n+1)=[k(x(n)+v(n))]\times T+v(n) \end{array}$$

similarly, the Euler discrete slave system can be formulated as:

(10) $$\begin{array}{l} x_{1}{(n+1)=[\mathrm{ay}}_{1}\left(n\right)-{\mathrm{bx}}_{1}{(n)+\mathrm{cy}}_{1}{(n)z}_{1}{(n)+y}_{1}{(n)z}_{1}{(n)w}_{1}\left(n)\right]\times {T+x}_{1}{(n)+u}_{1}\\ y_{1}{(n+1)=[\mathrm{dx}}_{1}{(n)+\mathrm{ly}}_{1}\left(n\right)-x_{1}{(n)z}_{1}\left(n\right)-{\mathrm{fv}}_{1}\left(n\right)-x_{1}{(n)z}_{1}{(n)w}_{1}\left(n)\right]\times {T+y}_{1}{(n)+u}_{2}\\ z_{1}(n+1)=[-{\mathrm{hz}}_{1}{(n)+y}_{1}{\left(n\right)}^{2}{+x}_{1}{(n)y}_{1}{(n)w}_{1}\left(n)\right]\times {T+z}_{1}{(n)+u}_{3}\\ w_{1}(n+1)=[-{\mathrm{jy}}_{1}{(n)z}_{1}\left(n\right)-{\mathrm{pw}}_{1}{(n)+x}_{1}{(n)y}_{1}{\left(n\right)}_{1}z_{1}\left(n)\right]\times {T+w}_{1}{(n)+u}_{4}\\ v_{1}{(n+1)=[k(x}_{1}{(n)+v}_{1}\left(n))\right]\times {T+v}_{1}{(n)+u}_{5} \end{array}$$

where T is the sampling period and T = 2 × 10^8^, x(n),y(n),z(n),w(n),v(n) represent state dynamics for the master system, and $x_{1}{(n),y}_{1}{(n),z}_{1}{(n),w}_{1}{(n),v}_{1}\left(n\right)$ represent state dynamics for the slave system, a, b, c, d, l, f, h, j, p, k are the parameters. The error dynamic system is obtained as:

(11) $$\begin{array}{l} \Delta e_{x}=T\times {[\mathrm{ae}}_{y}-{\mathrm{be}}_{x}{+c(y}_{1}{(n)z}_{1}\left(n\right)-{y(n)z(n))+y}_{1}{(n)z}_{1}{(n)w}_{1}\left(n\right)-{y(n)z(n)w(n)]+u}_{1}\\ \Delta e_{y}=T\times {[\mathrm{de}}_{x}{+\mathrm{le}}_{y}-{(x}_{1}{(n)z}_{1}\left(n\right)-x(n)z(n))-{\mathrm{fe}}_{v}-x_{1}{(n)z}_{1}{(n)w}_{1}{(n)+x(n)z(n)w(n)]+u}_{2}\\ \Delta e_{z}=T\times [-{\mathrm{he}}_{z}{+(y}_{1}{(n)+y(n))e}_{y}{+x}_{1}{(n)y}_{1}{(n)w}_{1}\left(n\right)-{x(n)y(n)w(n)]+u}_{3}\\ \Delta e_{w}=T\times [-{j(y}_{1}{(n)z}_{1}\left(n\right)-y(n)z(n))-{\mathrm{pe}}_{w}{+x}_{1}{(n)y}_{1}{(n)z}_{1}{(n)-x(n)y(n)z(n)]+u}_{4}\\ \Delta e_{v}=T\times {[k(e}_{x}{+e}_{v}{)]+u}_{5} \end{array}$$

where $e_{x}{\;=\;x}_{1}\left(n\right)-x(n)$, $e_{y}{\;=\;y}_{1}\left(n\right)-y(n)$, $e_{z}{\;=\;z}_{1}\left(n\right)-z(n)$, $e_{w}{\;=\;w}_{1}\left(n\right)-w(n)$, $e_{v}{\;=\;v}_{1}\left(n\right)-v(n)$, $u_{i}\left(i\;=\;1,2,3,4,5\right)$ is the synchronous controller. The target is to design a controller for master and slave systems such that the global synchronization holds. It means that the synchronization error converges to zero and stays in its vicinity:

(12) $$\underset{t\to \infty }{\lim}\left\|e(t)\right\|=0$$

**Theorem** **1.** *The master system (9) and the slave system (10) can be globally synchronized by the following controller:*

(13) $$\begin{array}{l} u_{1}=x(n)-x_{1}\left(n\right)-T\times {[\mathrm{ae}}_{y}{+\mathrm{cy}}_{1}{(n)z}_{1}\left(n\right)-{\mathrm{cy}(n)z(n)+y}_{1}{(n)z}_{1}{(n)w}_{1}\left(n\right)-y(n)z(n)w(n)]\\ u_{2}=y(n)-y_{1}\left(n\right)-T\times {[\mathrm{de}}_{x}{+2\mathrm{le}}_{y}-{x(n)z(n)+x}_{1}{(n)z}_{1}\left(n\right)-{\mathrm{fe}}_{v}+x(n)z(n)w(n)-x_{1}{(n)z}_{1}{(n)w}_{1}\left(n)\right]\\ u_{3}=z(n)-z_{1}\left(n\right)-T\times {[e}_{y}{(y}_{1}{(n)+y(n))+x}_{1}{(n)y}_{1}{(n)z}_{1}\left(n\right)-x(n)y(n)z(n)]\\ u_{4}=w(n)-w_{1}\left(n\right)-T\times \left[j(y(n)z(n\right)-y_{1}{(n)z}_{1}{(n))+x}_{1}{(n)y}_{1}{(n)z}_{1}\left(n\right)-x(n)y(n)z(n)]\\ u_{5}=v(n)-v_{1}\left(n\right)-T\times {[2\mathrm{ke}}_{v}{+\mathrm{ke}}_{x}] \end{array}$$

**Proof** **of** **Theorem** **1.** Considering the Lyapunov function as:

(14) $$V(e)=\frac{1}{2}{(e}_{x}^{2}{+e}_{y}^{2}{+e}_{z}^{2}{+e}_{w}^{2}{+e}_{v}^{2})\geq 0$$

derivative of (14) can be represented as:

(15) $$\begin{array}{c} \frac{\Delta V(e)}{T}{=e}_{x}\frac{{\Delta e}_{x}}{T}{+e}_{y}\frac{{\Delta e}_{y}}{T}{+e}_{z}\frac{{\Delta e}_{z}}{T}{+e}_{w}\frac{{\Delta e}_{w}}{T}{+e}_{v}\frac{{\Delta e}_{v}}{T}\\ \;{=e}_{x}{[\mathrm{ae}}_{y}-{\mathrm{be}}_{x}{+c(y}_{1}{(n)z}_{1}\left(n\right)-{y(n)z(n))+y}_{1}{(n)z}_{1}{(n)w}_{1}\left(n\right)-y(n)z(n)w(n)+\frac{u_{1}}{T}]\\ \;{+e}_{y}{[\mathrm{de}}_{x}{+\mathrm{le}}_{y}-{(x}_{1}{(n)z}_{1}\left(n\right)-x(n)z(n))-{\mathrm{fe}}_{v}-x_{1}{(n)z}_{1}{(n)w}_{1}(n)+x(n)z(n)w(n)+\frac{u_{2}}{T}]\\ \;{+e}_{z}[-{\mathrm{he}}_{z}{+(y}_{1}{(n)+y(n))e}_{y}{+x}_{1}{(n)y}_{1}{(n)w}_{1}\left(n\right)-x(n)y(n)w(n)+\frac{u_{3}}{T}]\\ \;{+e}_{w}[-{j(y}_{1}{(n)z}_{1}\left(n)-y(n)z(n)\right)-{\mathrm{pe}}_{w}{+x}_{1}{(n)y}_{1}{(n)z}_{1}\left(n\right)-x(n)y(n)z(n)+\frac{u_{4}}{T}]\\ \;{+e}_{v}{[k(e}_{x}{+e}_{v})+\frac{u_{5}}{T}] \end{array}$$

by substituting Equation (13) into Equation (15), $\frac{\Delta V(e)}{T}$ can be obtained as:

(16) $$\begin{array}{c} \frac{\Delta V(e)}{T}\;{=e}_{x}(-{\mathrm{be}}_{x}-\frac{e_{x}}{T}{)+e}_{y}(-{\mathrm{le}}_{y}-\frac{e_{y}}{T}{)+e}_{z}(-{\mathrm{he}}_{z}-\frac{e_{z}}{T}{)+e}_{w}(-{\mathrm{pe}}_{w}-\frac{e_{w}}{T}{)+e}_{v}(-{\mathrm{ke}}_{v}-\frac{e_{v}}{T})\\ \;=(-b-\frac{1}{T}{)e}_{x}^{2}+(-l-\frac{1}{T}{)e}_{y}^{2}+(-h-\frac{1}{T}{)e}_{z}^{2}+(-p-\frac{1}{T}{)e}_{w}^{2}+(-k-\frac{1}{T}{)e}_{v}^{2}<0 \end{array}$$

Since ΔV(e)/T is negative definite, the error dynamics system is globally asymptotically stable according to the Lyapunov stability theorem, and the master system and slave system errors will eventually converge to zero to reach full synchronization, regardless of the initial value. □

### 3.2. FPGA-Based Hyperchaotic Synchronization Design

**Figure 7:**
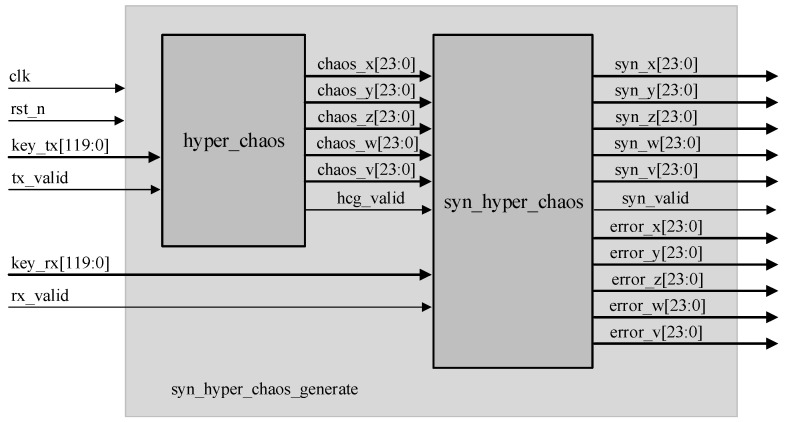
Top-level architecture of the hyperchaotic synchronization system.

Figure 7 provides the top-level architecture for the hyperchaotic synchronization system. To begin with, different 120-bit keys are input for the master and the slave system, and the intermediate signals, chaos_x [23:0], chaos_y [23:0], chaos_z [23:0], chaos_w [23:0], chaos_v [23:0], are generated by the hyper_chaos_generate module. All these are transmitted to the syn_hyper_chaos module, and the synchronized sequence and error signal are output under the operation of the non-linear feedback synchronization controller in the syn_hyper_chaos module, with the system reaching full synchronization when the error signal value is 0. The individual signal definition in Figure 7 is described in Table 5.

**Figure 8:**
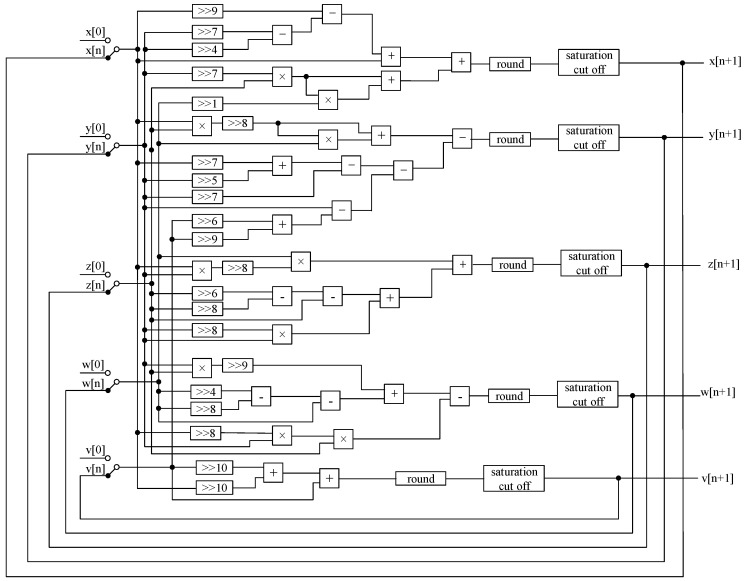
Algorithm flow diagram of the Master System.

**Figure 9:**
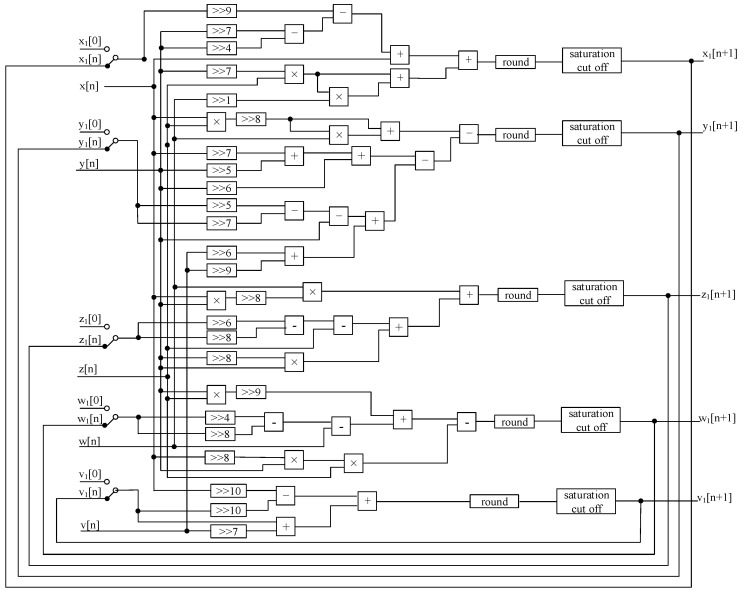
Algorithm flow diagram of the Slave System.

In order to prevent the multiplication result from overflowing, saturation truncation and rounding operations are required. In addition, the sign bit is also expanded to prevent data overflow when adding two data. In the hardware implementation, the multiplier consumes much more resource than the adder and subtractor. In order to reduce the number of multipliers, the parameters of the hyperchaotic system are obtained by shifting in this paper; the algorithm flow diagrams of the master system and the slave system are shown in Figure 8 and Figure 9, where “>>” indicates a right shift operation, “+” indicates an addition operation, and “−” indicates a subtraction operation, “×” for multiplication, “round” for rounding, “saturation cut off” means saturation cut-off.

The hyper_chaos module and the syn_hyper_chaos module use the Verilog HDL to develop a state machine to implement the above operations. The workflow of the hyper_chaos module state machine is shown below.

(1) The state machine is asynchronous reset, when the reset signal is valid, all signals are initialized, and the state converts to S0.
(2) S0: The initial key key_tx [119:0] is assigned to chaos_x [119:96], chaos_y [95:72], chaos_z [71:48], chaos_w [47:24], chaos_v [23:0], while the output valid signal is pulled up, indicating that the output is valid at the time, the state converts to S1.
(3) S1: Complete the shift operation and pull down the output valid signal, then the state converts to S2.
(4) S2: When the state converts to S2, the result of S1 is added and subtracted, and the result of the operation needs to be extended by one sign bit in order to prevent the overflow of the sum. This paper completes polynomial multiplication and fractional bit processing operations in the outside of the always block; first, the characteristics of the sign bit and the truncated part to determine the need of a carry bit—if the number is positive, the highest bit of the truncated part is 1—then it is necessary to generate a carry bit. If the number is negative, it is necessary to determine whether the highest bit of the truncated part and the other bits in addition to the highest bit have 1, if that situation exists it is not necessary to generate a carry bit. After calculating the carry bit, it is added to the number after the truncated decimal bit to complete the rounding operation, and at the same time, to prevent overflow when adding the carry bit, it is necessary to carry out a sign bit expansion, and then the state converts to S3.
(5) S3: In S2 we have completed the processing of fractional bits, in S3 we mainly deal with integer bits. We need to truncate the extra integer bits: if the part to be truncated and the highest bit after truncation is the same, that is, all 0 or all 1, then the part to be truncated is the extension of the sign bit, directly truncated; if different, the sign bit is judged, if positive, it will be changed to the maximum value that can be stored in the required format data, if negative, it will be changed to the minimum value that can be stored in the required format data. The final result is assigned to chaos_x, chaos_y, chaos_z, chaos_w, chaos_v, and then the valid signal of output is pulled up, the state converts to S1, the data is transferred to the syn_hyper_chaos system, and the set of data generation is completed.

The state machine workflow of the syn_hyper_chaos module is similar to that of the hyper_chaos and is shown below.

(1) The state machine is asynchronous reset, when the reset signal is valid, all signals are initialized and then the state converts to S0.
(2) S0: The initial key key_rx [119:0] is assigned to syn_x [119:96], syn_y [95:72], syn_z [71:48], syn_w [47:24], syn_v [23:0], the first output sequence is the initial key, in the second iteration, the output of the hyper_chaos module will be input to the syn_hyper_chaos module to participate in circular iteration, and then the output valid signal is pulled up, indicating that the output is valid at this time, and the state converts to S1.
(3) S1: To complete the shift operation and pull down the output valid signal, in the syn_hyper_chaos module, the output needs to add error signals, the value of the error signal is the difference between the syn signal and the chaos signal, at this point the valid signal of error is pulled up and then the state converts to S2.
(4) S2: When the state converts to S2, the result of S1 is added and subtracted, and the result of the operation needs to be extended by one sign bit in order to prevent the overflow of the sum. The same as the hyper_chaos module, the syn_hyper_chaos module completes polynomial multiplication and fractional bit processing operations in the outside of the always block. The valid signal of error is pulled down and the state converts to S3.
(5) S3: In S2 we have completed the processing of fractional bits, in S3 we deal mainly with integer bits. We need to truncate the extra integer bits and assign the final result to syn_x, syn_y, syn_z, syn_w, syn_v, the valid signal of output is pulled up, and then the state converts to S1, the set of data generation is finished.

## 4. Simulation Results

**Figure 10:**
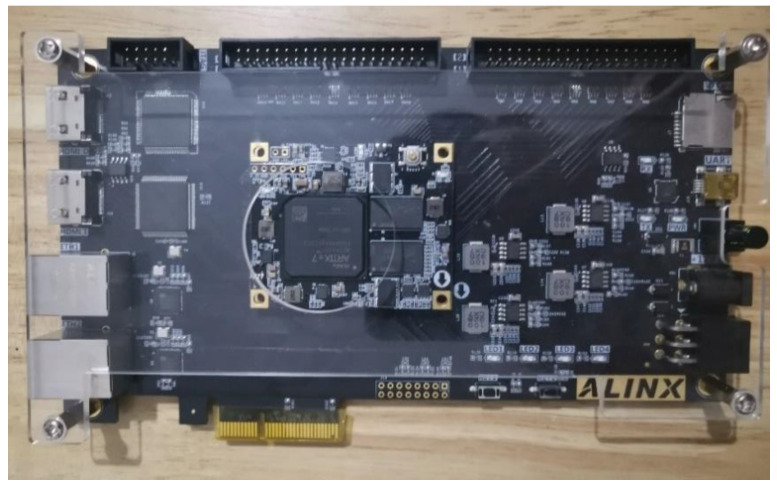
Xilinx Artix-7 Series AX7103 Development Board.

**Figure 11:**
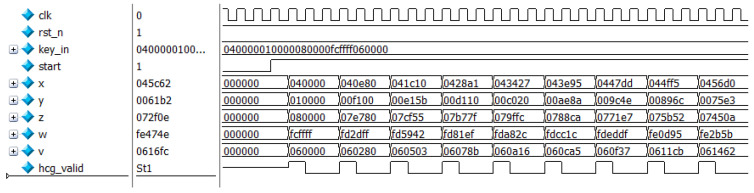
x, y, z, w, v time-series diagram for modelsim simulation.

**Figure 12:**
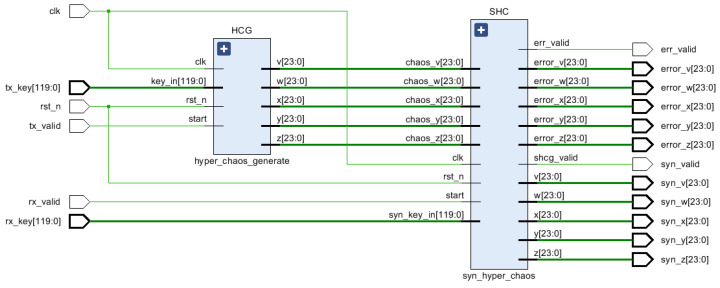
Chaotic synchronization RTL view.

The chip model chosen for this paper is Xilinx Airtex-7 series xc7a100tfgg484-2, as shown in Figure 10. The development board model used is the Xilinx Artix-7 series AX7103 development board. Simulation is completed by vivado2019.1 and modelsim2017.4. The simulation results of the new 5-dimensional hyperchaotic system based on FPGA are shown in Figure 11. The initial key is set as 120′h040000_ 010000_080000_FCFFFF_060000. Compared with MATLAB simulation data, both results are consistent. It indicates that the result of the digital design of the FPGA-based chaotic system is accurate. Furthermore, it takes three clock cycles to generate a valid data which greatly improves the efficiency of the system compared with the contents of the references. Meanwhile, the modelsim simulation data were exported and the corresponding NIST tests performed, and the results are shown in Table 6. The test results show that the chaotic sequences generated by the digitized hyperchaotic system still have high randomness. The RTL view of the chaotic synchronous top-level design is shown in Figure 12. 

**Figure 13:**
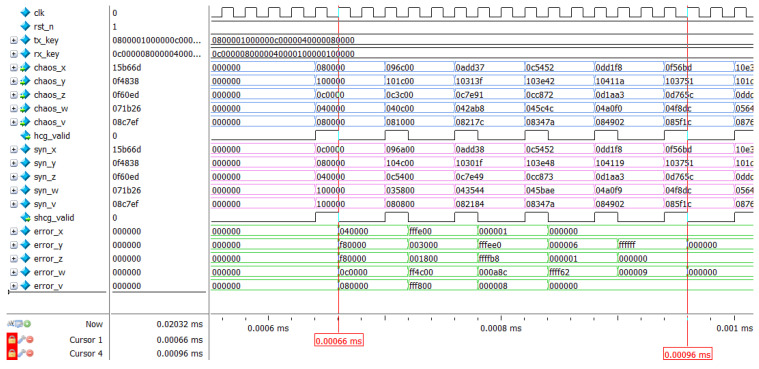
RTL view of chaotic synchronization.

**Figure 14:**
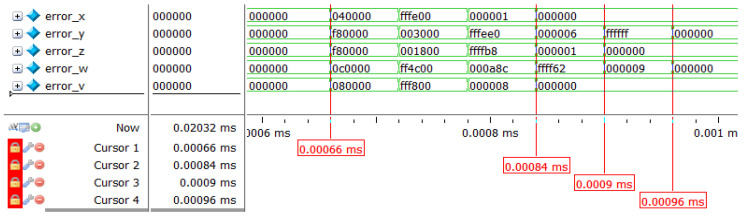
Partial view of chaotic synchronization results simulated with modelsim.

By observing Figure 12, it can be seen that Figure 12 corresponds to the top-level design in Section 3. Table 7 shows the resource consumption and the result of the FPGA-based synchronization design of the hyperchaotic system is shown in Figure 13, Figure 14 provides a partial view of the simulation results. It can be determined that the output time of the first synchronization sequence is 0.00066 ms under the 50 MHz clock condition, and when 0.00096 ms, error_x [23:0], error_y [23:0], error_z [23:0], error_w [23: 0], and error_v [23:0] are all zero, achieving complete synchronization of two hyperchaotic systems with the same structure and different initial values, with a very minimal establishment time, and thus demonstrating the superior performance of hardware implementation of hyperchaotic synchronization.

**Figure 15:**
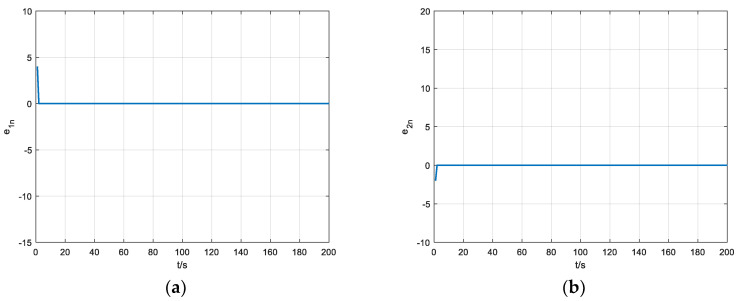

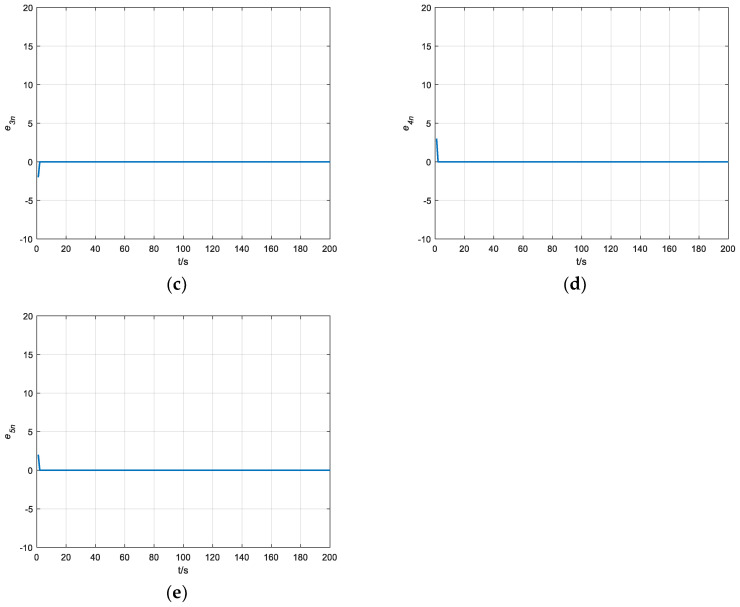
Error result of MATLAB simulation. (**a**) Diagram of errorx simulation result, (**b**) diagram of errory simulation result, (**c**) diagram of errorz simulation result, (**d**) diagram of errorw simulation result, (**e**) diagram of errorv simulation result.

To show the design results more clearly, in this paper, the modelsim simulation data of the chaotic synchronous system are exported to generate .dat files and input into MATLAB R2018 to generate the final error result graph as shown in Figure 15.

It can be concluded form Figure 15 that the error of each output sequence soon reaches 0, indicating that the above design is correct. Compared with the software implementation of chaos synchronization, the hardware implementation has high stability and does not show significant fluctuations in the error convergence process. Due to the high-speed performance of the hardware itself, the FPGA-based chaotic synchronization time is much shorter than the software-based chaotic synchronization time. Compared with the content of the same field, the overall convergence speed of the synchronous controller designed in this paper is rapid, indicating that at the same clock rate, the synchronous controller only needs a few iterations to achieve full synchronization, which demonstrates the excellent performance of the FPGA-based chaotic synchronous design.

## 5. Conclusions

In this paper, a new five-dimensional chaotic system suitable for synchronization was constructed and its phase diagrams observed by MATLAB simulation. The analysis of the Lyapunov exponent spectrum and bifurcation diagram shows that the chaotic system has hyperchaotic characteristics, and the maximum Lyapunov exponent is 1.9380 with a favorable permutation entropy index. A new five-dimensional hyperchaotic system based on FPGA was designed and implemented. Simulation results show that the FPGA-based hyperchaotic system designed in this paper can generate a chaotic sequence in three clock cycles. In addition, the synchronization of the hyperchaotic system was also investigated. A multivariate nonlinear feedback synchronization controller was proposed, and the synchronization control of the high-dimensional hyperchaotic system with higher order terms was designed and implemented based on FPGA. The simulation result shows that two hyperchaotic systems with the same structure and different initial values can be synchronized quickly under the effect of the controller, which proves the validity of the designed controller.

## Figures and Tables

**Table 1:** Lyapunov exponents and the corresponding attractor morphology.

k	LE_1_	LE_2_	LE_3_	LE_4_	LE_5_	Attractor Morphology
−0.095	0.0137	−0.0810	−0.9829	−5.0398	−5.0774	Periodic attractor
0.055	0.4044	0.0064	−0.1136	−1.4236	−10.9422	Chaotic attractor
0.423	1.9380	0.1391	−0.0084	−2.3637	−11.1328	Hyperchaotic attractor

**Table 2:** Comparison of the maximum Lyapunov exponent.

Proposal	The Maximum Lyapunov Exponent
ours	1.9380
Ref. [25]	1.0461
Ref. [26]	0.7362
Ref. [27]	1.0100
Ref. [28]	0.0044
Ref. [29]	1.0241

**Table 3:** NIST test of proposed 5−D hyperchaotic system.

Testing Item	*p*-Value(x)	Result
Approximate Entropy	0.210398	pass
Block Frequency	0.180283	pass
Cumulative Sum	0.582341	pass
FFT	0.596701	pass
Frequency	0.645639	pass
Linear Complexity	0.151631	pass
Longest Run	0.408543	pass
NonOverlapping template	0.601890	pass
Overlapping template	0.851142	pass
Random Excursion	0.621039	pass
Random Excursions Variant	0.602752	pass
Rank	0.298427	pass
Runs	0.054103	pass
Serial1	0.200412	pass
Serial2	0.145922	pass
Universal	0.304519	pass

**Table 4:** Comparison of permutation entropy value.

Hyperchaotic System	m	t	PE
Ours	3	1	0.7024
Ref. [28]	3	1	0.6841
Ref. [29]	3	1	0.5714
Ref. [30]	3	1	0.6201

**Table 5:** Signal definition.

Signal	Signal Definition
clk	The system clock
rst_n	The reset signal
key_tx [119:0]	Initial key on the transmitter
tx_valid	Transmit initial key valid signal
key_rx [119:0]	Initial key on the receiver
rx_valid	Receive initial key valid signal
syn_x/syn_y/syn_z/ syn_w/syn_ v [23:0]	The synchronised sequence
error_x/error_y/error_z/ error_w/error_v [23:0]	Sequence error

**Table 6:** NIST test results of the digitized chaotic sequence.

Testing Item	*p*-Value(x)	Result
Approximate Entropy	0.352142	pass
Block Frequency	0.199847	pass
Cumulative Sum	0.421255	pass
FFT	0.751245	pass
Frequency	0.604212	pass
Linear Complexity	0.320412	pass
Longest Run	0.201485	pass
Non-Overlapping template	0.581245	pass
Overlapping template	0.782121	pass
Random Excursion	0.604712	pass
Random Excursions Variant	0.580073	pass
Rank	0.300047	pass
Runs	0.067581	pass
Serial1	0.200412	pass
Serial2	0.102476	pass
Universal	0.294578	pass

**Table 7:** Table of resource consumption.

Resource	Utilization	Available	Utilization%
LUT	2477	41,000	6.04
LUTRAM	26	13,400	0.19
FF	1963	82,000	2.39
DSP	40	240	16.67

## Data Availability

All results and data obtained can be found in open access publications.
